# Identification of an Inflammatory Response-Related Gene Signature to Predict Survival and Immune Status in Glioma Patients

**DOI:** 10.1155/2022/8972730

**Published:** 2022-05-18

**Authors:** Zhaoyue Yan, Yushuai Gao, Jinliang Yu, Zhiyuan Shen, Xingyao Bu

**Affiliations:** Department of Neurosurgery, Henan Provincial People's Hospital, People's Hospital of Zhengzhou University, Zhengzhou 450003, China

## Abstract

**Background:**

Glioma is the most common primary brain tumor with high mortality and poor outcomes. As a hallmark of cancers, inflammatory responses are crucial for their progression. The present study is aimed at exploring the prognostic value of inflammatory response-related genes (IRRGs) and constructing a prognostic IRRG signature for gliomas.

**Materials and Methods:**

We investigated the relationship between IRRGs and gliomas by integrating the transcriptomic data for gliomas from public databases. Differentially expressed IRRGs (DE-IRRGs) were identified in the GSE4290 cohort. Further, univariate, least absolute shrinkage and selection operator, and multivariate Cox regression analyses were conducted to construct an IRRG signature using The Cancer Genome Atlas (TCGA) cohort. Gliomas from the Chinese Glioma Genome Atlas (CGGA) cohort were employed for independent validation. The performance of gene signature was assessed by survival and receiver operating characteristic curve analyses. The differences in clinical correlations, immune infiltrate types, immunotherapeutic response predictions, and pathway enrichment among subgroups were investigated via bioinformatic algorithms.

**Results:**

In total, 37 DE-IRRGs were determined, of which 31 were found to be associated with survival. Ultimately, eight genes were retained to construct an IRRG signature that further classified glioma patients into two groups; the high-risk group suffered a poorer outcome as compared to the low-risk group. Furthermore, the high-risk group was significantly correlated with several risk factors, including older age, higher tumor grade, IDH wild type, 1p19q noncodel, and MGMT unmethylation. The nomogram was constructed by integrating the risk scores and other independent clinical characteristics. Moreover, the high-risk group had a greater immune infiltration and was most likely to benefit from immunotherapy. Gene set enrichment analysis suggested that immune and oncogenic pathways were enriched in high-risk glioma patients.

**Conclusion:**

We constructed a signature composed of eight IRRGs for gliomas, which could effectively predict survival and guide decision-making for treatment.

## 1. Introduction

Gliomas are the most prevalent type of primary intracranial tumors, representing 80% of all brain neoplasms [1]. Globally, approximately 1,000,000 new patients are diagnosed with gliomas, annually [2]. Although gliomas constitute less than 2% of all new cases of diagnosed cancers, these are often associated with substantial mortality rates in patients [3]. According to the World Health Organization (WHO) classification, 2021, gliomas are categorized into lower-grade glioma (LGG, grade 2-3) and glioblastoma (GBM, grade 4) [4, 5]. Despite recent advances in treatment options, including surgery, postoperative radiotherapy, and chemotherapy, the prognoses for gliomas remain poor. The median survival time for GBM is less than 15 months due to the extensive intracranial invasion and aggressiveness [6, 7]. In contrast, the patients with LGG exhibit relatively favorable prognoses, up to a median survival time of approximately 7 years [8]. Unfortunately, the cases of LGG inevitably progress to higher grades and develop resistance against the treatment [9]. Although current molecular markers, for example, dehydrogenase (IDH) mutations and 1p/19q codeletion, have been used for molecular pathological diagnoses, clinical treatment, and prognostic evaluation [10, 11], several researchers have tried developing therapeutic strategies by targeting these markers, only a few advances have been made to date in clinical practice [12]. Therefore, it is necessary to identify novel biomarkers for effectively predicting clinical prognoses and therapeutic responses in glioma.

Inflammatory responses have been recognized as the hallmarks of cancer [13]. Studies have confirmed that inflammation plays an integral role in tumor initiation, angiogenesis, and metastases [13, 14]. An estimated 15–20% of all cancer-related deaths, worldwide, are attributed to the potential infections and inflammatory responses [14, 15]. The persistent noncontrollable local and systemic inflammatory responses induced by tissue damage may mediate the initiation and development of cancers [16]. In the recent decade, an increasing number of studies on the intersection between inflammation and cancer pathogenesis show that immune cells, especially those involved in innate immune responses, play key roles in the progression of multiple cancers [13, 17]. Indeed, inflammation is present at earlier stages of tumor growth, even before the detection of malignancy, resulting in the development of a microenvironment facilitating precancerous lesions to result in cancer [13, 14, 18]. In addition, previous studies report that inflammation can accelerate the development of heightened malignancy by inducing the activities of the nearby cancerous cells due to the release of cytokines and overproduction of reactive oxygen species [19, 20]. Mostofa et al. report that multiple inflammatory mediators including cytokines, cyclooxygenases, STAT3, NF-*κ*B, and oxidative stress are closely associated with glioma progression [21]. However, the knowledge on the relationships between inflammatory response-related genes (IRRGs) and the outcomes in patients with glioma is limited.

To examine and annotate the integrative functions of IRRGs in gliomas, RNA-sequencing data and clinical information were extracted from public databases. Differentially expressed IRRGs (DE-IRRGs) between normal and glioma tissues were identified using the Gene Expression Omnibus (GEO) database. Next, we constructed a prognostic IRRG signature to predict clinical outcomes of glioma using the data from The Cancer Genome Atlas (TCGA). The performance of the signature was further validated using an independent cohort from the Chinese Glioma Genome Atlas (CGGA) database. Moreover, analyses of clinical correlation, immune infiltrates, immunotherapeutic response prediction, and pathway enrichment were further performed. In summary, we constructed a prognostic signature based on IRRGs to predict prognoses in gliomas, and the findings may provide a reliable foundation for prognostic assessment and the development of personalized targeted treatment strategies.

## 2. Materials and Methods

### 2.1. Data Sources

A total of 200 IRRGs were identified in the Molecular Signatures (MSigDB) database (http://www.gsea-msigdb.org/gsea/msigdb) [22, 23]. The microarray GSE4290 cohort for differential gene analysis by Sun et al. [24] was downloaded from the GEO database (https://www.ncbi.nlm.nih.gov/), comprising 23 normal brain and 176 glioma tissues. To obtain deeper insights, a whole-genome microarray analysis was performed for the total cellular RNA using the Affymetrix Human Genome U133 Plus 2.0 Array. Using the “limma” R package [25], differentially expressed genes (DEGs) between normal and tumor tissues were identified with false discovery rate (FDR) < 0.05 and a ∣log_2_ fold change (FC) | >1. Venn diagrams for the commonly shared DE-IRRGs between IRRGs and DEGs were drawn using VennDiagram. A protein-protein interaction (PPI) network was generated using the STRING database (https://string-db.org/) [26]. Correlation analysis was used to assess the relationship among these genes in R. Gene Ontology (GO) and Reactome pathway enrichment analyses were utilized to annotate biological processes, cellular components, molecular functions, and the underlying pathways using WebGestalt (http://www.webgestalt.org/) [27]. Furthermore, we downloaded the RNA-sequencing expression profiles and corresponding clinical data from two databases, including the LGG and GBM cohorts from TCGA database (https://portal.gdc.cancer.gov/) and the two Chinese cohorts (the 693 and 325 cohorts) from the CGGA database (http://www.cgga.org.cn/). The samples with missing clinical information were filtered out. In total, 1471 glioma and 23 normal brain samples were acquired. The detailed clinicopathological features of glioma are summarized in Table 1.

### 2.2. Construction and Validation of a Prognostic IRRG Signature

The data from TCGA were utilized to construct the prognostic signature, and the data from the CGGA cohorts were used for validation. First, the univariate Cox regression analysis was performed to detect the prognostic DE-IRRGs based on TCGA data. Only the overall survival- (OS-) associated genes (*P* < 0.05) were further analyzed. Subsequently, the least absolute shrinkage and selection operator (LASSO) regression analysis was used to obtain an optimal predictive model by minimizing the risk of overfitting [28]. Finally, a prognostic signature was constructed using the coefficients derived from multivariate Cox regression analysis, using the following formula: risk score = ∑_*i*=1_^*n*^Coefficient(*i*) × Expression(*i*), where Coefficient(*i*) represents the regression coefficient for each gene and Expression(*i*) represents the gene expression level. This formula was used for calculating risk scores for each patient in both TCGA and ICGC cohorts. Principal component analysis (PCA) was used to identify the distribution of different groups. The survival curves were analyzed using the Kaplan–Meier method, and log-rank tests were used to assess the significance. Moreover, the receiver operating characteristics (ROC) curves were evaluated to validate the accuracy of the signature. The signature was also validated in the CGGA dataset.

### 2.3. GEPIA, cBioPortal, and DiseaseMeth Databases

Gene Expression Profiling Interactive Analysis (GEPIA, http://gepia.cancer-pku.cn/) database is a webserver to analyze the expression data from RNA sequencing through the standard analysis pipeline [29]. Using this database, we verified the prognostic significance of genes constituting the IRRG signature.

The cBio Cancer Genomics Portal database (cBioPortal database) (http://cbioportal.org) comprises the data of more than 5000 tumor tissues, thereby enabling multilevel analyses for multiple tumors [30]. Mutation data and copy number variants of these genes were retrieved from this database. Mutation data and copy number variants of these genes were retrieved using this database.

Human Disease Methylation Database Version 2.0 (DiseaseMeth2.0, http://bio-bigdata.hrbmu.edu.cn/diseasemeth/), a human disease methylation database, is used to analyze the DNA methylation level in several diseases [31]. The differences in DNA methylation between normal and glioma tissues were compared as described previously.

### 2.4. Survival and Clinical Correlation Analysis of the IRRG Signature

The stratified survival analyses for gliomas were performed using clinical characteristics, including age, gender, grade, IDH, radiotherapy, chemotherapy, 1p19q, and MGMT. The survival outcomes in two groups were described for all patients stratified in groups through the Kaplan–Meier curves. The relationships between clinical characteristics and risk score were analyzed both in TCGA and CGGA cohorts.

### 2.5. Nomogram Construction

Univariate and multivariate Cox proportional hazard regression analyses were employed to identify the independent prognostic values of the signature as well as other clinicopathological factors. We constructed a nomogram to estimate survival probabilities using the independent prognostic indicators from TCGA database based on Cox proportional hazard regression models [32]. The nomogram was constructed using the “rms” package in R [33]. The calibration curve was plotted to evaluate the performance of the nomogram. ROC curve analysis was used to assess the discriminative performance of the nomogram.

### 2.6. Single-Sample Gene Set Enrichment Analysis (ssGSEA) and Immune Response Analysis

To examine the differences in immune cell infiltrations and functions of our signature, 29 immune infiltration-associated gene sets were obtained from Bindea et al. [34]. Next, we performed ssGSEA to calculate the scores for every gene set in each patient using the “GSVA” R package [35]. The tumor purity, immune, stromal, and ESTIMATE scores for each glioma sample were calculated using the “Estimate” R package as described previously [36]. The above microenvironment compositional parameters were compared between the high- and low-risk groups. In addition, the relationship between the risk score and expression of the immune checkpoints was investigated by Spearman's correlation analysis. In this study, the immunophenoscore (IPS) was used to evaluate the responses to anti-CTLA4 and anti-PD1 treatment regimens using The Cancer Immunome Atlas (TCIA, https://tcia.at/) database [37].

### 2.7. Protein Network Construction and Gene Set Enrichment Analyses (GSEA)

GeneMANIA is a flexible interactive web server for performing network analysis for protein-protein interactions (PPIs) and predicting the functions of preferred genes [38]. In the present study, we used this web tool to generate and analyze the interactions among these eight genes and identify other potential binding partners (at default set value of 20) in the regulatory network. GO and KEGG analyses were used to annotate the putatively affected pathways.

To further elucidate the potential mechanisms underlying the IRRG signature-suggested poor outcomes in gliomas, GSEA was performed using the GSEA software [39]. The reference gene sets (c5.go.bp.v7.2.symbols.gmt, c2.cp.kegg.v7.1.symbols.gmt, and h.all.v7.4.symbols.gmt) were obtained from the MSigDB database [23]. The significant enrichments were calculated and filtered with *P* < 0.05 and FDR < 0.05 as the threshold.

### 2.8. Statistical Analysis

The variables were compared using Student's *t*-test, chi-square test, the Wilcoxon rank-sum test, and the Mann–Whitney *U* test, as appropriate. The log-rank test was performed for estimating the significance of the Kaplan–Meier analysis. *P* < 0.05 was considered statistically significant. All statistical analyses were performed using R.

## 3. Results

### 3.1. Identification of Prognostic DE-IRRGs

**Figure 1:**
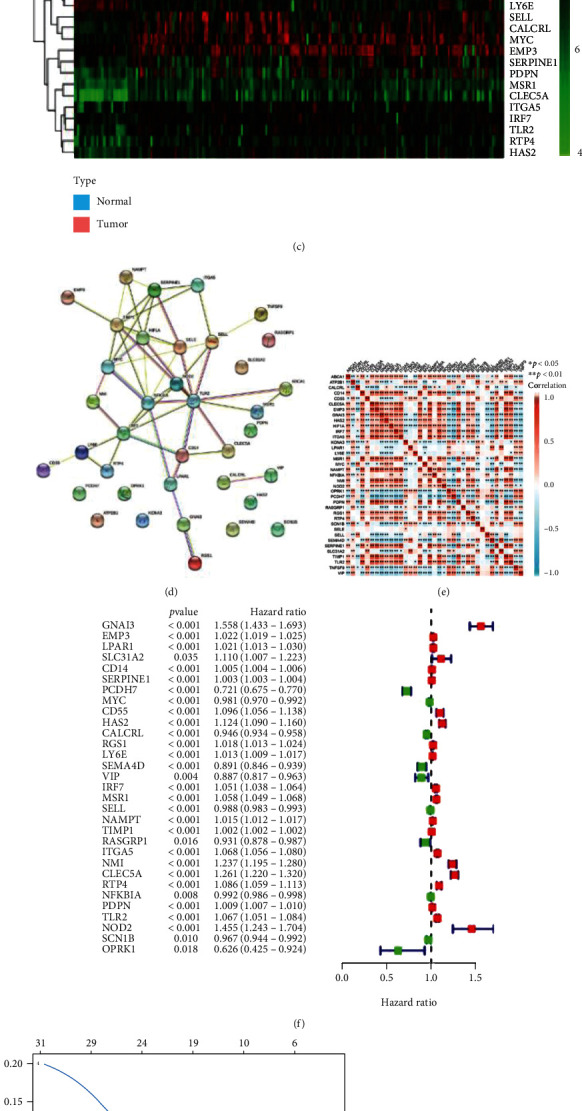
Identification of target genes and construction of an IRRG signature. (a) Volcano plot for differentially expressed genes (DEGs) between 23 normal brain tissues and 176 glioma tissues in the GSE4290 cohort. (b) Venn diagram depicting the intersection (*n* = 37) of DEGs and inflammatory response-related genes (IRRGs). (c) The expression profiles of 37 differentially expressed IRRGs (DE-IRRGs). (d) Protein-protein interaction (PPI) network showing the interactions among 37 DE-IRRGs genes. (e) The correlation network of 37 DE-IRRGs genes by Spearman analysis. (f) Univariate Cox regression analysis to determine 31 prognostic DE-IRRGs (*P* < 0.05) in TCGA cohort. (g) LASSO regression analysis for further screening out the 11 candidate genes. (h) Illustration of the LASSO coefficient spectrum for the 11 candidate genes. (i) Multivariate Cox regression analysis identifies 8 target genes. ^∗^*P* < 0.05, ^∗∗^*P* < 0.01, and ^∗∗∗^*P* < 0.001.

The workflow of this study is shown in Supplemental Figure 1. We compared the differences in gene expressions between 23 normal brain tissues and 176 glioma tissues in the GSE4290 cohort. According to the criteria, FDR < 0.05 and ∣log_2_FC  | >1.0, a total of 2216 DEGs were identified, including 826 upregulated and 1390 downregulated genes in glioma samples from GSE4290 (Figure 1(a)). In addition, MSigDB an online database was used to identify 200 IRRGs. The Venn diagram, as shown in Figure 1(b), represents the 37 commonly shared DE-IRRGs between DEGs and IRRGs. The expression levels of 37 DE-IRRGs were visualized using a heat map (Figure 1(c)). The PPI network of these genes was constructed using the STRING database (Figure 1(d)). A significant correlation between the majority of the DE-IRRGs was obtained using Pearson's correlation matrix (Figure 1(e)). Out of the 37 DE-IRRGs, a total of 31 prognostic genes were screened by univariate Cox regression in TCGA cohort (Figure 1(f)). GO analysis was performed to annotate these genes, and the results of Reactome enrichment analysis showed that these 31 genes were significantly related to the diseases of the immune system and those associated with the TLR signaling cascade (Supplemental Figure 2).

### 3.2. Construction of a Prognostic IRRG Signature in TCGA Cohort

To construct an IRRG-based prognostic signature to predict the risk of gliomas, the 31 prognostic DE-IRRGs were further analyzed. First, we conducted a LASSO regression analysis for these 31 genes to avoid overfitting for the risk signature. As a result, 11 candidate genes were retained for the subsequent analysis (Figures 1(g) and 1(h)). Next, multivariate Cox regression analysis was performed for these candidates. Eventually, a total of 8 target genes (GNAI, EMP3, PCDH7, CALCRL, TIMP1, ITGA5, NMI, and NFKBIA) and their coefficients were employed to construct the predictive signature (Table 2). The IRRG signature was computed as described: risk score = GNAI∗0.1132 + EMP3∗0.0086–PCDH7∗0.1125–CALCRL∗0.0212 + TIMP1∗0.0008 + ITGA5∗0.2136 + NMI∗0.0504–NFKBIA∗0.0126. The forest map showed that GNAI, EMP3, TIMP1, ITGA5, and NMI were the risk factors, while PCDH7, CALCRL, and NFKBIA showed opposite trends (Figure 1(i)). In addition, we examined the influence of these 8 target genes on the prognoses of gliomas using the GEPIA website. High GNAI, EMP3, TIMP1, ITGA5, and NMI while low PCDH7, CALCRL, and NFKBIA expressions could lead to poor prognoses in glioma patients. These results were in line with our previous results (Supplemental Figure 3). Subsequently, we also utilized the information from 15 diverse glioma datasets to investigate the mutational landscape of these 8 genes. The results showed that EMP3 and ITGA5 were most commonly genomically altered (Supplemental Figure 4) and may be involved in glioma progression. Considering the effects of methylation associated with gene expressions in glioma, especially the IDH1 status, we evaluated the methylation levels of these signature-related genes using the DiseaseMeth2.0 database. The results showed that the methylation levels of ITGA5 (a risk gene) were lower in both LGG and GBM tissues relative to the normal tissues (Supplementary Table 1).

**Figure 2:**
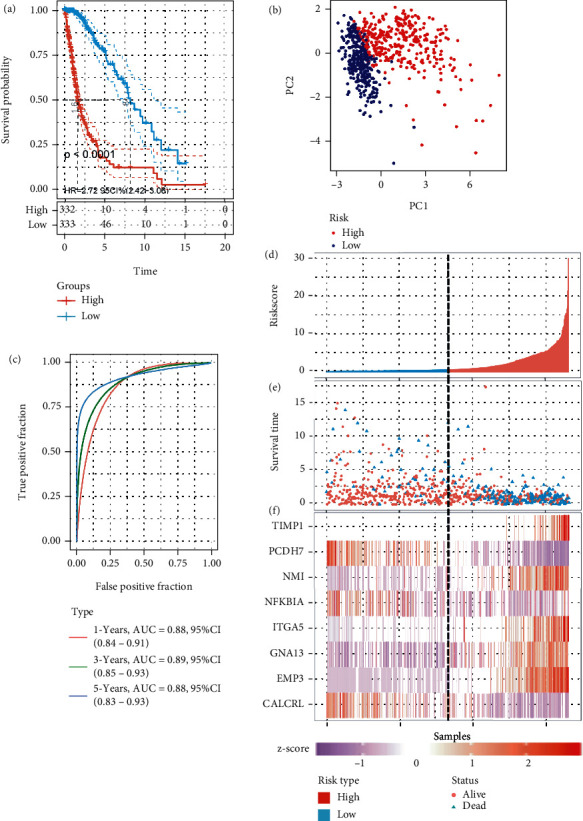
Prognostic analysis using the IRRG signature in TCGA cohort. (a) Kaplan–Meier curves for OS prediction in the low- and high-risk groups in TCGA cohort. (b) Principal component analysis (PCA) shows the separation between low- and high-risk groups in TCGA cohort. (c) The prognostic value of the IRRG signature at 1, 3, and 5 years via time-dependent ROC curve analysis in TCGA cohort. (d) The risk score distribution for each patient in TCGA cohort. (e) The distribution of survival time and survival status in TCGA cohort. (f) The heat map shows that GNAI, EMP3, TIMP1, ITGA5, and NMI exhibit high expression levels, whereas PCDH7, NFKBIA, and CALCRL show low expression levels in TCGA cohort.

The risk scores of individual patient were computed with the above formula. The glioma samples were assigned into high- and low-risk groups with the median risk score value (0.685) of TCGA cohort. Kaplan–Meier plots indicated that patients with high-risk score suffered a markedly worse OS than patients who have low-risk score (Figure 2(a)). The result of PCA showed that patients in low- and high-risk groups were divided in diverse directions (Figure 2(b)). Time-dependent ROC curve analyses were performed to assess the performance of IRRG signature for the prognostic prediction of patients with glioma. The areas under the curve (AUC) of ROC were 0.88, 0.89, and 0.88 at 1, 3, and 5 years, respectively (Figure 2(c)). The distribution of survival status is shown in Figure 2(d). In addition, the scatter plot demonstrated that the mortality rates in the high-risk group were higher (Figure 2(e)). The heat map suggested that GNAI, EMP3, TIMP1, ITGA5, and NMI exhibited high expression levels, whereas PCDH7, CALCRL, and NFKBIA show low levels of expression (Figure 2(f)).

### 3.3. Evaluation of the IRRG Signature Using the CGGA Validation Cohort

**Figure 3:**
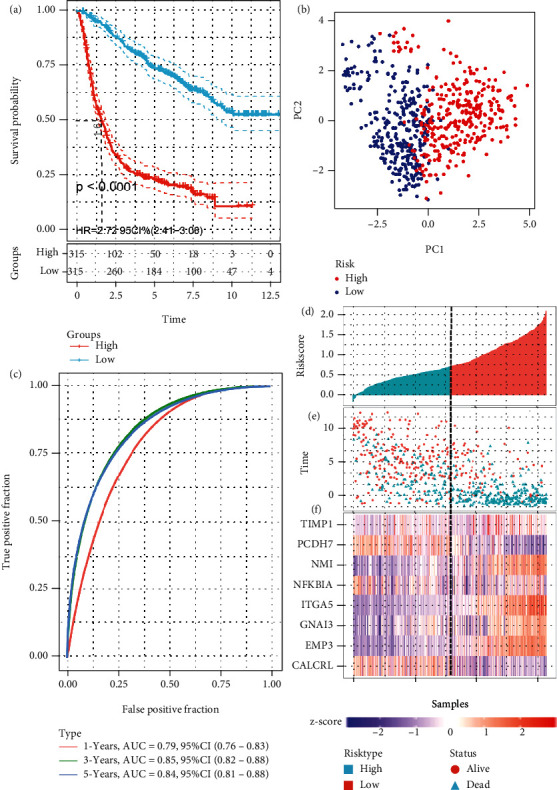
Prognostic analysis using the IRRG signature in the CGGA cohort. (a) Kaplan–Meier curve for OS prediction in the low- and high-risk groups in the CGGA cohort. (b) PCA shows the separation between low- and high-risk groups in the CGGA cohort. (c) The prognostic values of the IRRG signature at 1, 3, and 5 years via time-dependent ROC curve analysis in the CGGA cohort. (d) The risk score distribution for each patient in the CGGA cohort. (e) The distribution of survival time and survival status in the CGGA cohort. (f) The heat map shows that GNAI, EMP3, TIMP1, ITGA5, and NMI exhibit high expression levels, whereas PCDH7, NFKBIA, and CALCRL show low expression levels in the CGGA cohort.

Next, we investigated the feasibility of using the IRRG signature for predicting outcomes using the CGGA cohort. These patients were also sorted into low- or high-risk groups according to the median risk score calculated using the formula described above. Similarly, Kaplan–Meier curves indicated the patients at high risk showed an obviously worse survival than their low-risk counterparts (Figure 3(a)). PCA revealed a complete separation between two groups (Figure 3(b)). Moreover, the AUCs were 0.79, 0.85, and 0.84 at 1, 3, and 5 years, respectively (Figure 3(c)). The risk plot for CGGA comprising the risk score ranking, survival status according to the gliomas, and the gene expression heat map is illustrated in Figures 3(d)–3(f). The results obtained in the CGGA cohort followed the trends observed in TCGA cohort.

It was relevant to show more prognostic abilities of the eight-gene signature in GBM. Therefore, we further analyzed the Kaplan–Meier and ROC curves of GBM patients in both TCGA and CGGA datasets. The Kaplan–Meier survival curves for TCGA-GBM and CGGA-GBM samples demonstrated significant survival differences between the low- and high-risk groups based on the best optimal cutoff value from the log-rank test (Supplemental Figures 5(a) and 5(b)). As shown, the AUC values at 5 years for the TCGA-GBM and CGGA-GBM samples were 0.57 and 0.66, respectively (Supplemental Figures 5(c) and 5(d)). The above results further validated the stability of the IRRG signature.

With the development of sequencing technology, many glioma cancer prognostic gene signatures have been recently published. We compared our signature with those reported in five previously published studies by performing a ROC analysis. As shown in Supplemental Figure 6, the AUC of our signature for determining the 5-year OS was higher as compared to the five other reports in both TCGA and CGGA datasets, which implied that our signature has better performance for predicting prognoses. Taken together, our results confirmed that the IRRG signature showed a more accurate and stable evaluation of prognoses for patients with glioma.

### 3.4. Relationship of the IRRG Signature with Survival Prognosis and Clinical Features

**Figure 4:**
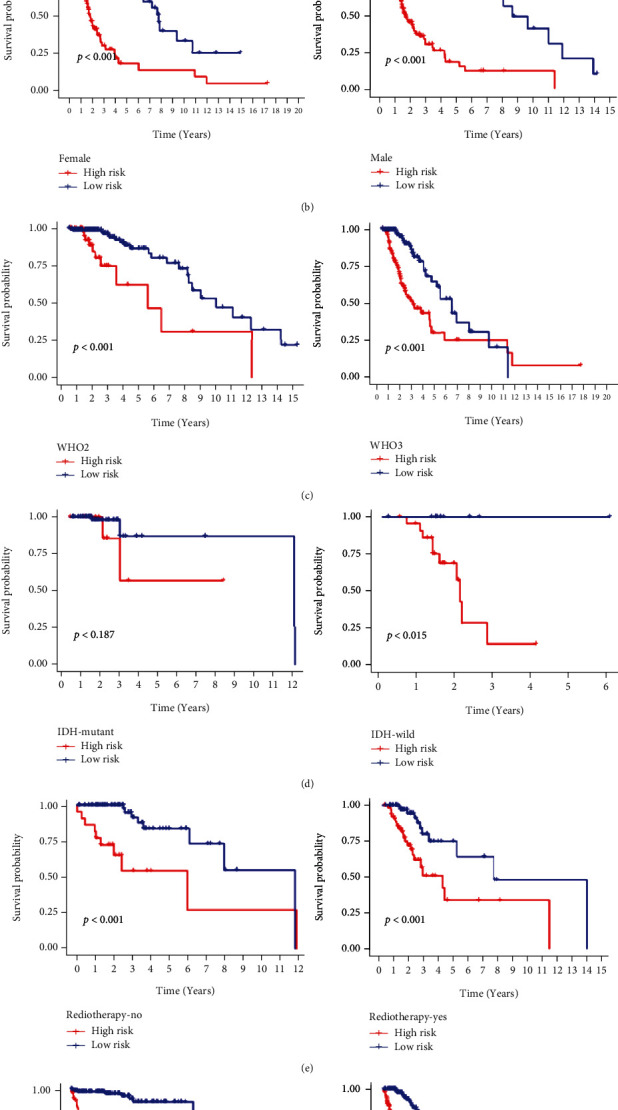
Kaplan–Meier survival curves stratified by different clinical features in TCGA cohort. Survival analyses using the IRRG signature in patients with different (a) ages, (b) gender, (c) grade, (d) IDH, (e) radiotherapy, and (f) chemotherapy.

**Figure 5:**
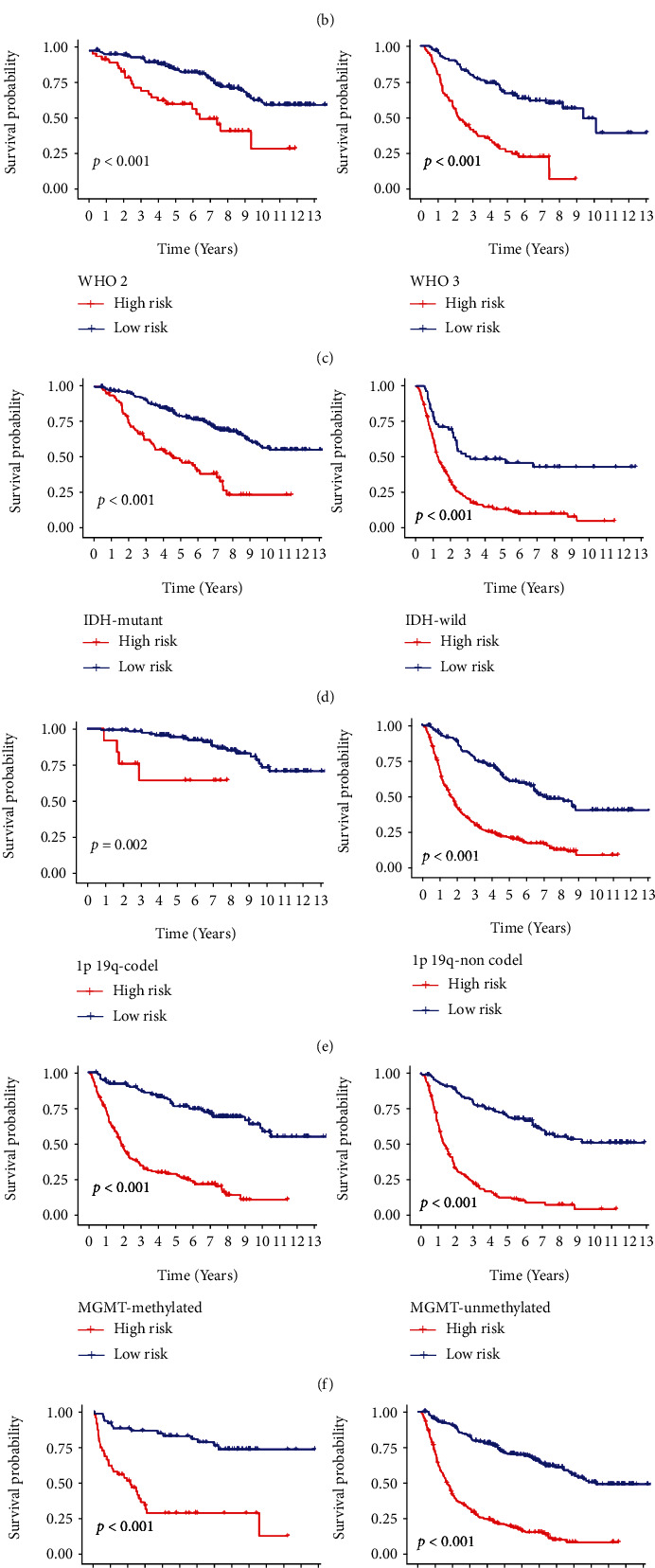
Kaplan–Meier survival curves stratified by different clinical features in the CGGA cohort. Survival analyses using the IRRG signature in patients with different (a) age, (b) gender, (c) grade, (d) IDH, (e) 1p19q status, (f) MGMT methylation, (g) radiotherapy, (h) and chemotherapy.

To investigate a potential prognostic value of the IRRG signature in stratified cohorts, glioma patients were divided according to their age (≤45 or >45), gender (female or male), grade (WHO 2, 3, or 4), IDH (mutation or wild type), radiotherapy (no or yes), chemotherapy (no or yes), 1p19q (codel or noncodel), and MGMT (methylated or unmethylated) status. In TCGA dataset, the Kaplan–Meier curves suggested that the high-risk group showed shorter OS relative to the low-risk group in all stratified subgroups, except for the IDH mutation subgroup (Figures 4(a)–4(f)). Surprisingly, all gliomas of grade 4 belonged to the high-risk group. Similar findings were noticed in the CGGA database for all stratified subgroups, including the radiotherapy and chemotherapy subgroups (Figure 5(a)–5(h)). These results demonstrated that classifications using the IRRG signature could precisely determine patients with poor survival regardless of their clinical parameters.

**Figure 6:**
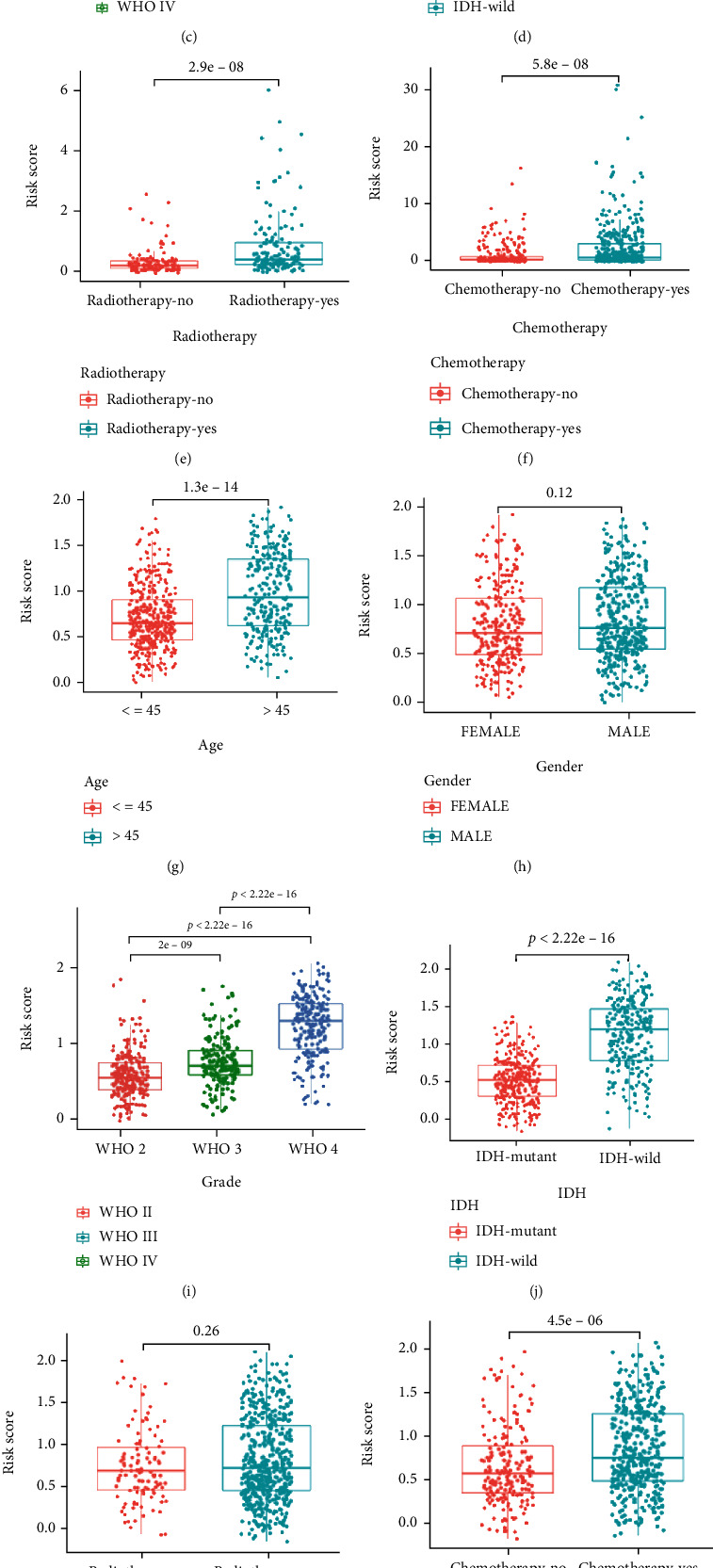
The risk score in different subgroups is divided by clinical features in TCGA and CGGA cohorts. Survival analyses using IGGR signature in patients with different (a, g) ages, (b, h) gender, (c, i) grade, (d, j) IDH, (e, k) radiotherapy, (f, l) chemotherapy, (m) 1p19q status, and (n) MGMT methylation.

By investigating the associations between the IRRG signature and various clinical features of gliomas, we found significant positive associations of the risk score with age (Figures 6(a) and 6(g)) and grade (Figures 6(c) and 6(i)) both in TCGA and CGGA cohorts. Next, we observed that the high-risk group was significantly associated with IDH wild type (Figures 6(d) and 6(j)) and chemotherapy (Figures 6(f) and 6(l)) in the two cohorts and were the poor prognostic factor for gliomas. In addition, the risk score was significantly higher in 1p19q noncodel (Figure 6(m)) and MGMT unmethylated (Figure 6(n)) subgroups in the CGGA database. The differences in risk scores between the with and without radiotherapy subgroups were evident in TCGA (Figure 6(e)) but not in the CGGA dataset (Figure 6(k)). No differences were found based on the gender in the two datasets (Figures 6(b) and 6(h)).

### 3.5. Construction and Evaluation of the Nomogram

**Figure 7:**
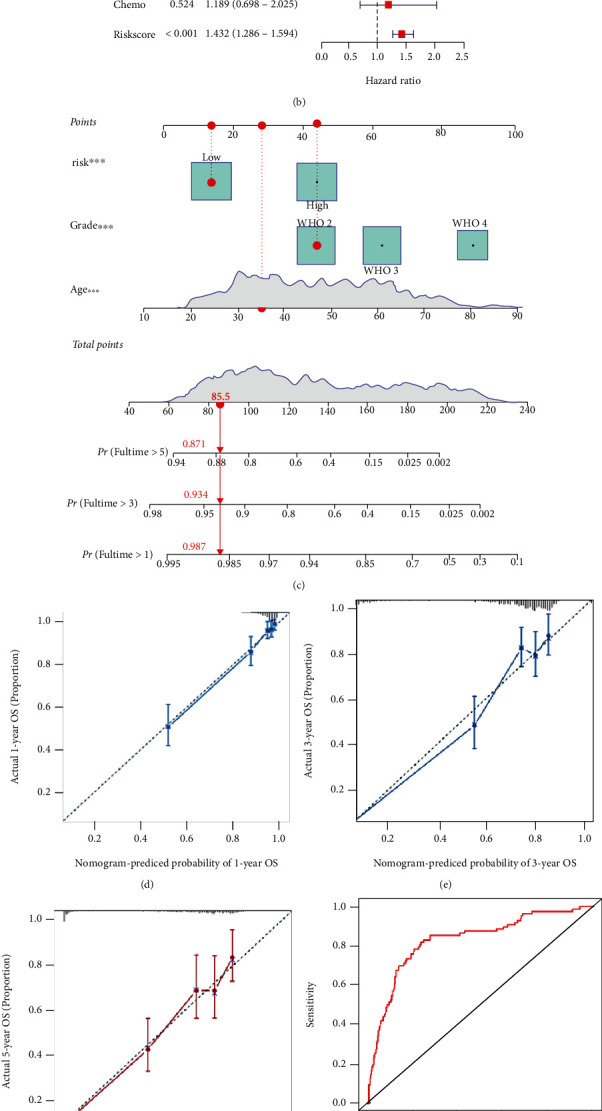
The nomogram for prognostic probability prediction in TCGA database. (a) Univariate and (b) multivariate Cox regression analyses for the IRRG signature and clinical factors. (c) Construction of the nomogram for predicting survival probability for glioma patients at 1, 3, and 5 years. (d–f) The calibration curves for the nomogram at 1-, 3-, and 5-year survival. (g–i) Time-dependent receiver operating characteristic (ROC) curves for the nomogram for predicting the 1-, 3-, and 5-year OS. ^∗∗∗^*P* < 0.001.

To identify the independence of the IRRG signature, we performed a Cox analysis for the signature both in TCGA and CGGA cohorts. In TCGA cohort, the results from the univariate Cox analysis showed that age, grade, chemotherapy status, and risk score were closely related to the OS (all *P* < 0.05) (Figure 7(a)). These factors were then included in multivariate Cox analysis, and the results demonstrated that age, grade, and risk were independent prognostic parameters for gliomas (Figure 7(b)). Analogous results were obtained in the CGGA cohort (Supplemental Figure 7). In TCGA dataset, based on the weight of each selected independent prognostic clinical variable in the Cox regression analysis (Supplementary Table 2), a prognostic nomogram was constructed to offer a quantitative approach for clinicians to estimate the survival rates for patients with gliomas (Figure 7(c)). The calibration plots showed better consistency between the predictions by the nomogram and actual observation for 1-, 3-, and 5-year survival probabilities (Figures 7(d)–7(f)). The time-dependent ROC curve analyses were used to evaluate the accuracy of the nomograms; the AUCs for the 1-, 3-, and 5-year OS predictions for the nomogram were 0.832, 0.838, and 0.823, respectively (Figures 7(g)–7(i)).

### 3.6. IRRG Signature to Predict Immune Infiltration and Responses to Immune Checkpoint Inhibitors (ICIs)

**Figure 8:**
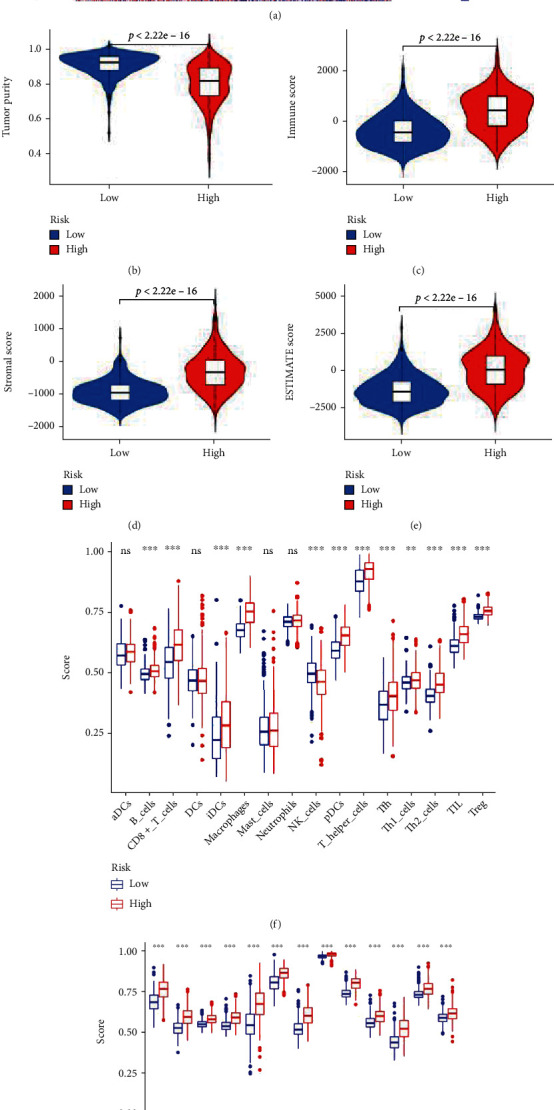
The immune status between different risk groups as analyzed in patients with glioma based on TCGA database. (a) The heat map shows the scores of 29 immune-associated gene sets between low- and high-risk groups by ssGSEA arithmetic. The violin plots display the differences in (b) tumor purity, (c) ESTIMATE scores, (d) immune scores, and (e) stromal scores between low- and high-risk groups. (f) The expressions of 16 immune cells between low- and high-risk groups. (g) The expressions of 13 immune-related functions between low- and high-risk groups. ^∗∗^*P* < 0.01 and ^∗∗∗^*P* < 0.001; ns: no significance.

Subsequently, we examined whether the signature was related to the tumor microenvironment (TME) using the IRRG signature construed based on the IRRGs. The enrichment scores for diverse immune cells, functions, and pathways in each glioma sample were quantized by ssGSEA using 29 immune gene sets. The high-risk group represented a relatively high immune status (Figure 8(a)). Specifically, through the ESTIMATE algorithm, the tumor purity was found to be lower in the high-risk group, indicating higher immune cell infiltration in the tumor tissues (Figure 8(b)). As expected, the high-risk group was associated with higher immune, stromal, and ESTIMATE scores as compared to the low-risk group (Figures 8(c)–8(e)). Additionally, as compared to the low-risk group, the composition of several immune cells was found to be more abundant in the high-risk group (Figure 8(f)). All immune functions or pathways in the high-risk group were also upregulated (Figure 8(g)). The IRRG signature was associated with the TME and the immune system of glioma patients with high-risk scores.

**Figure 9:**
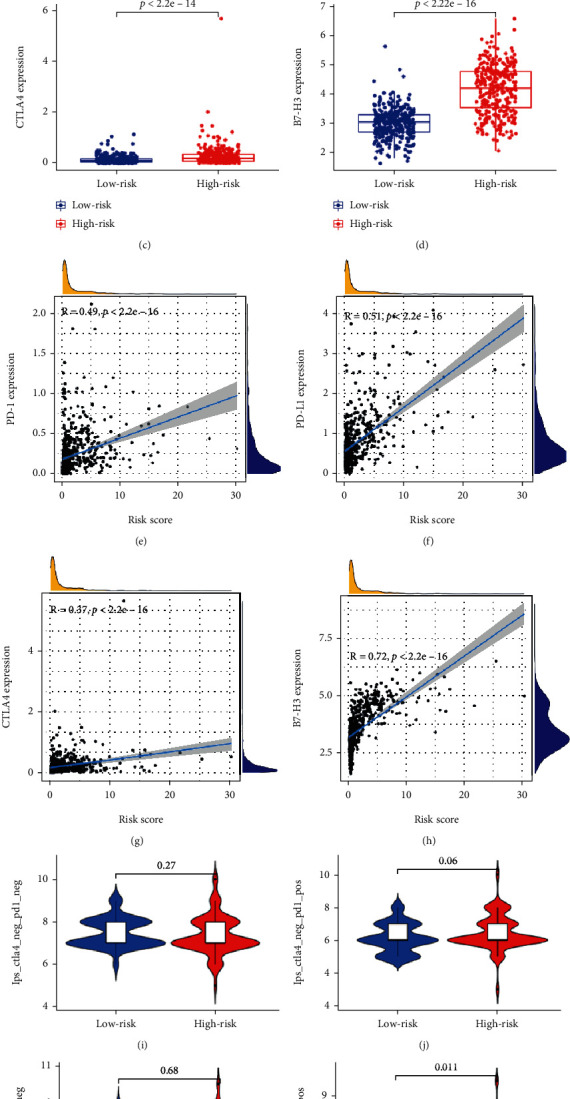
The relationship between the IRRG signature and the levels of ICI expressions. The comparison of the levels of expression of (a) PD-1, (b) PD-L1, (c) CTLA4, and (d) B7-H3 between the low- and high-risk groups. The Pearson correlation coefficients between risk scores and (e) PD-1, (f) PD-L1, (g) CTLA4, and (h) B7-H3. Comparison of the immunophenoscore (IPS) between the low- and high-risk groups stratified as (i) CTLA4^negative^/PDL1^negative^, (j) CTLA4^negative^/PDL1^positive^, (k) CTLA4^positive^/PDL1^negative^, and (l) CTLA4^positive^/PDL1^positive^ treatments.

We then examined the correlation between risk scores and ICIs (PD1, PDL1, CTLA4, and B7-H3) in gliomas. Patients in the high-risk group tended to show higher levels of ICI expressions relative to those in the low-risk group (Figures 9(a)–9(d)). In addition, a positive relationship between risk scores and expression levels of the ICIs was observed (Figures 9(e)–9(h)). We then specifically investigated the impact of the risk scores for evaluating the immunotherapeutic outcomes using TCIA database. The results showed that the diverse groups showed different immunogenicities as responses to CTLA4^positive^/PD1^positive^ treatments (Figures 9(i)–9(l)). These findings suggested differential effects of immune status on treatment using ICIs, and the higher was the immune infiltration, the greater was the intensity of immunosuppression.

### 3.7. IRRG Signature-Related Biological Functions and Pathways

**Figure 10:**
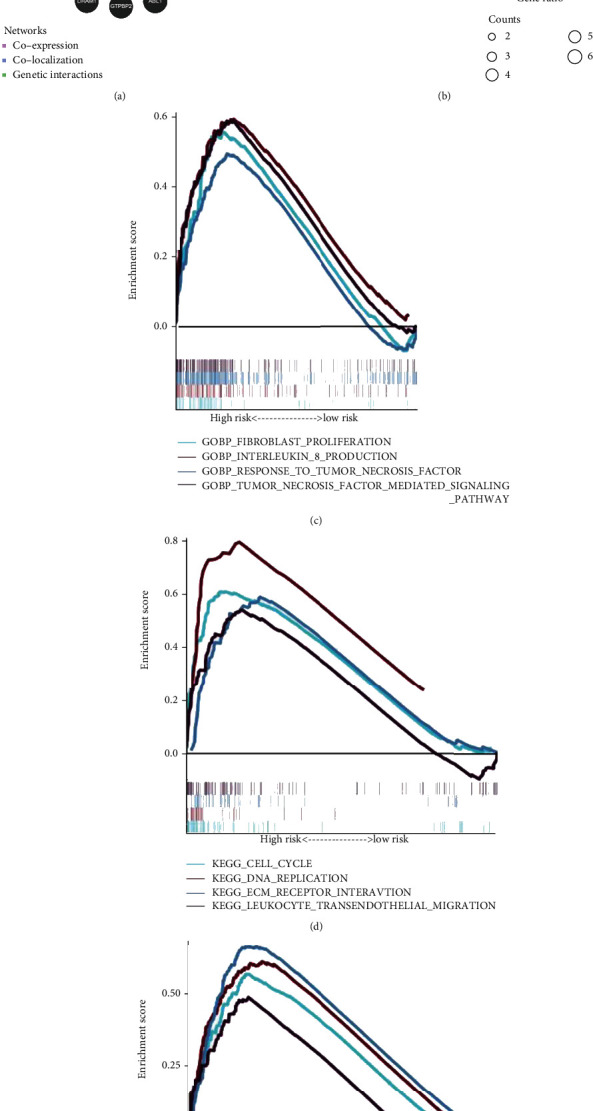
IRRG signature-related biological functions and enriched pathways. (a) The regulatory network involving eight genes and 20 potential binding proteins was constructed using the GeneMANIA database. (b) GO and KEGG analyses for 28 genes. GSEA shows the significantly enriched processes and pathways in (c) Gene Ontology analysis, (d) Kyoto Encyclopedia of Genes and Genomes analysis, and (e) Hallmark gene set analysis based on the IRRG signature.

We constructed an interaction network to investigate the possible relationships among these eight genes using GeneMANIA. Additional 20 potential binding partners were automatically identified (Figure 10(a)). We then performed GO and KEGG pathway enrichment analyses for these 28 genes. In terms of KEGG enrichment, these genes were enhanced in the TNF signaling pathway, NF−kappa B signaling pathway, and Chagas disease (Figure 10(b)).

To analyze the potential mechanisms leading to poor outcomes in gliomas, we performed GSEA based on the IRRG signature using TCGA data. GO analysis showed that fibroblast proliferation, interleukin-8 production, response to TNF, and TNF-mediated signaling pathway were the significantly enriched biological processes in the high-risk group (Figure 10(c)). Moreover, the results of KEGG enrichment indicated malignancy-related pathways were most active in the high-risk group, such as the cell cycle, DNA replication, and leukocyte transendothelial migration (Figure 10(d)). The hallmark gene sets demonstrated that the high-risk group was markedly correlated with DNA repair, epithelial-mesenchymal transition, interferon-gamma response, and PI3K/Akt/mTOR signaling (Figure 10(e)).

## 4. Discussion

Glioma is the lethal brain tumor which often results in an undesirable prognosis [40]. The 5-year survival rate of GBM is a disappointing 4.7% owing to its highly aggressive nature [9]. Conventional treatments including surgery, postoperative radiotherapy, and chemotherapy yield unsatisfactory survival outcomes. However, currently, there are no effective targeted molecular therapies for gliomas that can improve the curative effects. Increasing data support a driving role of the inflammatory microenvironment in tumorigenesis, including colorectal cancer and hepatocellular carcinoma [22, 41]. Michelson et al. show that aberrant signaling induced by inflammation is involved in the malignant transformation of LGG [42]. Therefore, knowledge of the biological functions and mechanisms underlying inflammation is of significance to help predict prognosis and treatment responses in glioma patients.

In this study, we systematically analyzed the genomic landscape and prognostic value of 200 IRRGs in patients with glioma. According to the LASSO-Cox regression model, a prognostic signature consisting of eight IRRGs (GNAI, EMP3, PCDH7, CALCRL, TIMP1, ITGA5, NMI, and NFKBIA) was constructed and validated in the CGGA database. The patients in the low-risk group tended to show better OS in the two datasets. Furthermore, we found that the high-risk group was obviously correlated with several risk factors, such as older age, higher tumor grades, IDH wild type, 1p19q noncodel, and MGMT unmethylation status. This conclusion was in agreement with that of a previous study [43]. Subsequently, the IRRG signature was proven as a ponderable prognostic feature independent of other clinical parameters. Further, we identified the independent prognostic indicators (age, grade, and risk scores) and constructed the nomogram to accurately predict the 1-, 3-, and 5-year survival probabilities, which may be of help for improving the individualized treatment strategies for gliomas. Overall, these results indicated that the IRRG signature was significantly associated with prognoses of glioma patients.

The prognostic signature was constructed based on eight IRRGs, including GNAI, EMP3, PCDH7, CALCRL, TIMP1, ITGA5, NMI, and NFKBIA. The mRNA and protein expressions of GNAI were upregulated in human glioma samples and cell lines. Liu et al. show that the downregulation of miR-200a can activate AKT and promote glioma cell proliferation by upregulating GNAI in human glioma [44]. EMP3 can promote tumor growth along with activation of TGF-*β* signaling in intracranial GBM xenografts [45]. Moreover, Neftel et al. by single-cell RNA-seq demonstrate that EMP3 levels are high in GBM-infiltrating macrophages [46]. PCDH7 is known to be downregulated in cervical cancer tissues and cell lines as compared to their corresponding controls, and the overexpression of PCDH7 markedly inhibits the migration and invasion abilities of cervical cancer cells [47]. Expression of CALCRL is undetected in the pancreatic cancer cells while primary human pancreatic stellate and endothelial cells express CALCRL [48]. The high expression of TIMP1 confers poor prognoses in several types of cancers including GBM. Moreover, the effects of topoisomerase inhibitors in GBM decrease due to the upregulation of TIMP-1 [49]. Chen et al. show that NEAT1 promotes the expression of ITGA5 in glioma tissues by competitively binding to miR-128-3p. Specifically, ITGA5 may promote the growth of glioma cells via the FAK signaling pathway without decomposition by miR-128-3p [50]. NMI is reportedly upregulated in glioma tissues and may play an important role in the development of GBM through inflammatory responses [51]. In another previous study using a glioma mouse model, higher expression of NMI is even more frequently detected in glioma tissues as compared to the adjacent nonneoplastic brain tissues, thus confirming the diagnostic utility [52]. Moreover, NFKBIA can serve as a promising prognostic marker for those with an advanced grade of gliomas [53]. In the present study, GNAI, EMP3, TIMP1, ITGA5, and NMI were found to be significant risk factors, while PCDH7, CALCRL, and NFKBIA were substantial protective factors. Collectively, the expression patterns and prognostic values of IRRGs for patients with glioma in our research were according to those of the previous studies.

In recent years, the use of immunotherapy has made noticeable breakthroughs in the treatment of various types of cancers [54]. However, patients with gliomas have not substantially benefited from immunotherapy. Therefore, we further investigated the potential impacts of the IRRG signature on the immune microenvironment. In this study, patients in the high-risk category from TCGA cohort showed an obviously lower tumor purity and higher immune scores as compared to those in the low-risk group. Previous studies show that high immunity is closely related to a poor prognosis in patients with gliomas [43, 55]. Infiltration of immunosuppressive cells, mainly the macrophages and Tregs, plays a critical role in the efficacy of immunotherapy and tumor immune evasion. Recent investigations show that the presence of macrophages and Treg cells is involved in the unfavorable prognosis owing to their functions in immune invasion [56]. The patient with high-risk score in the present study possessed higher fractions of macrophages and Treg cells. This may also explain why the prognoses of patients in the high-risk group were poor. Additionally, we analyzed the association between the risk score and ICIs, and the results showed that the expressions of PD1, PD-L1, CTLA4, and B7-H3 in the high-risk group were significantly higher, that is, these patients were most likely to benefit from immunotherapy. The present findings were in strong agreement with those of previous studies [22, 55].

Finally, GSEA was performed to investigate the underlying biological functions and mechanisms by which the IRRG signature affected the prognoses. The results suggested that inflammation-associated biological processes including interleukin-8 production and response to TNF were markedly enriched in the high-risk group, thus further confirming the close relationship between inflammatory responses and tumor progression. In addition, enhanced activities in extensive malignancy-related pathways were involved in the high-risk populations. For example, a recent study by Song et al. demonstrates that PLAC2 may play tumor-suppressive roles in an RPL36-dependent manner and block the cell cycle by downregulating the expression of CDK2 [57]. Knocking down WNK3 can inhibit the invasion of glioma cell lines by regulating the epithelial-mesenchymal transition, especially in hypoxic microenvironments [58]. Therefore, we reasonably speculated that the enhancement of these prooncogenic signaling pathways likely underlies the worse outcomes in the high-risk group. However, further experiments are required to elucidate their specific mechanisms in gliomas.

There were some limitations to this study. First, the data used were not generated by us but were collected from various public repositories. Information on the clinical symptoms of some patients with glioma was lacking. Second, the constructed signature in the present study requires multicenter data for in-depth verification. Third, our study was a retrospective design that might contain various types of bias, like selection bias and information bias. Therefore, we added this content to our manuscript. Finally, our findings still require experimental validation in the future.

## 5. Conclusion

In conclusion, we examined the expression profiles and prognostic values of IRRGs in patients with glioma and constructed a prognostic signature consisting of eight IRRGs. The IRRG signature was verified to accurately classify patients with diverse survival outcomes both in TCGA and CGGA cohorts. Notably, the prognostic signature was further confirmed to be of great clinical significance for the analyses of clinical correlation, immune infiltrates, pathway enrichment, and immunotherapeutic response predictions. These findings may deepen our understanding of gliomas induced by inflammatory responses and shed light on potential prognostic biomarkers and therapeutic targets for gliomas.

## Figures and Tables

**Table 1:** Clinicopathological characteristics of glioma patients included in this study.

Variables	TCGA cohort	CGGA cohort	GSE4290
Age			
≤45	328	370	—
>45	337	260	
Gender			
Male	383	373	
Female	282	257	—
Tissue			
Normal	0	0	23
Tumor	665	630	176
Grade			
2	245	222	12
3	261	189	31
4	159	219	77
IDH			
Wild	34	282	—
Mutation	91	310	—
N/A	540	38	—
1p/19q			
Codel	—	135	—
Noncodel	—	432	—
N/A	—	63	—
MGMT			
Methylated	—	288	—
Unmethylated	—	257	—
N/A	—	85	—
Radiotherapy			
Yes	143	503	—
No	118	111	—
N/A	404	16	—
Chemotherapy			
Yes	403	408	—
No	262	199	—
N/A	—	23	—

Abbreviations: TCGA: The Cancer Genome Atlas; CGGA: Chinese Glioma Genome Atlas; IDH: isocitrate dehydrogenase; MGMT: O6-methylguanine-DNA-methyltransferase; N/A: not available.

**Table 2:** Coefficients of the eight target IRRGs from the multivariate Cox regression analysis.

Gene	Coefficient	HR (95% CI)	*P* value
GNAI3	0.1132	1.1198 (1.0005-102535)	0.048977
EMP3	0.0086	1.0086 (1.0025-1.0138)	0.000858
PCDH7	-0.1125	0.8935 (0.8297-0.9234)	0.002951
CALCRL	-0.0212	0.9790 (0.9790-0.9654)	0.003053
TIMP1	0.0008	1.0008 (1.0003-1.0013)	0.000481
ITGA5	0.02136	1.0215 (1.0002-1.0434)	0.047966
NMI	0.0504	1.0517 (0.9946-1.1121)	0.076774
NFKBIA	-0.0126	0.9874 (0.9875-0.9813)	8.21*e* − 05

## Data Availability

The data of this study are available in GEO database (https://www.ncbi.nlm.nih.gov/), TCGA database (https://portal.gdc.cancer.gov/), and CGGA database (http://www.cgga.org.cn/).

## Supplementary Materials

Supplementary Materials Supplemental Figure 1: flowchart of the study design. Supplemental Figure 2: functional analysis for 31 differentially expressed inflammatory response-related genes using the WebGestalt database. (A) Gene Ontology (GO) enrichment analysis. GO terms consist of molecular functions (MF), biological processes (BP), and cellular components (CC). (B) Reactome pathway analysis. Supplemental Figure 3: the prognostic analysis of eight target IRRG in the signature using the GEPIA database. (A) GNAI. (B) EMP3. (C) PCDH7. (D) CALCRL. (E) TIMP1. (F) ITGA5. (G) NMI. (H) NFKBIA. Supplemental Figure 4: the oncoprint plot describes the mutations, copy number variations, and structural variant landscape for the eight target IRRGs in glioma. Supplemental Figure 5: prognostic analysis for the IRRG signature in GBM patients in both TCGA and CGGA cohorts. (a) Kaplan–Meier curve for OS prediction in GBM patients in TCGA cohort. (b) The prognostic value of the IRRG signature at 5 years via ROC curve analysis in TCGA cohort. (c) Kaplan–Meier curve for OS prediction for GBM patients in the CGGA cohort. (d) The prognostic value for the IRRG signature at 5 years via ROC curve analysis in the CGGA cohort. Supplemental Figure 6: time-dependent ROC analysis for comparing the six signatures (including the IRRG signature of the present study) in predicting 5-year OS in both TCGA and CGGA databases. Supplemental Figure 7: univariate (a) and multivariate (b) Cox regression analyses of the IRRG signature and clinical factors.
