# Supplementary material for: Identification of an Inflammatory Response-Related Gene Signature to Predict Survival and Immune Status in Glioma Patients
Source: J Immunol Res. 2022 May 18;2022:8972730. doi: 10.1155/2022/8972730 (PMC9132661; doi:10.1155/2022/8972730)
Supplement: Supplementary Materials — Supplemental Figure 1: flowchart of the study design. Supplemental Figure 2: functional analysis for 31 differentially expressed inflammatory response-related genes using the WebGestalt database. (A) Gene Ontology (GO) enrichment analysis. GO terms consist of molecular functions (MF), biological processes (BP), and cellular components (CC). (B) Reactome pathway analysis. Supplemental Figure 3: the prognostic analysis of eight target IRRG in the signature using the GEPIA database. (A) GNAI. (B) EMP3. (C) PCDH7. (D) CALCRL. (E) TIMP1. (F) ITGA5. (G) NMI. (H) NFKBIA. Supplemental Figure 4: the oncoprint plot describes the mutations, copy number variations, and structural variant landscape for the eight target IRRGs in glioma. Supplemental Figure 5: prognostic analysis for the IRRG signature in GBM patients in both TCGA and CGGA cohorts. (a) Kaplan–Meier curve for OS prediction in GBM patients in TCGA cohort. (b) The prognostic value of the IRRG signature at 5 years via ROC curve analysis in TCGA cohort. (c) Kaplan–Meier curve for OS prediction for GBM patients in the CGGA cohort. (d) The prognostic value for the IRRG signature at 5 years via ROC curve analysis in the CGGA cohort. Supplemental Figure 6: time-dependent ROC analysis for comparing the six signatures (including the IRRG signature of the present study) in predicting 5-year OS in both TCGA and CGGA databases. Supplemental Figure 7: univariate (a) and multivariate (b) Cox regression analyses of the IRRG signature and clinical factors. [file 8972730.f1.docx]

Supplementary Table 1. Testing the nomogram by the Cox regression analysis.

| Variable | Coefficient | HR | S.E. | Wald Z | *P* |
| --- | --- | --- | --- | --- | --- |
| Risk score | 0.032 | 1.033 | 0.007 | 4.606 | 4.11E-06 |
| Grade | 1.132 | 3.101 | 0.005 | 9.624 | 0 |
| Age | 0.023 | 1.023 | 0.118 | 4.348 | 1.38E-05 |

Supplementary Table 2. The methylation levels of these signature-related genes between disease (LGG and GBM) and normal tissues using DiseaseMeth2.0 database.

| Gene | Genomic region | Transcript | LGG | | GBM | |
| --- | --- | --- | --- | --- | --- | --- |
|  |  |  | p-value | MeanMethyl  (Disea-Normal) | p-value | MeanMethyl  (Disea-Normal) |
| CALCRL | chr2:188312521-188315021 | NM_005795 | <0.05 | -0.299 | >0.05 | -0.072 |
| EMP3 | chr19:48826628-48829128 | NM_001425 | <0.05 | 0.222 | >0.05 | -0.063 |
| GNAI3 | chr1:110089185-110091685 | NM_006496 | <0.05 | -0.042 | >0.05 | 0.001 |
| ITGA5 | chr12:54812550-54815050 | NM_002205 | <0.05 | -0.061 | <0.05 | -0.019 |
| NFKBIA | chr14:35873460-35875960 | NM_020529 | <0.05 | -0.034 | >0.05 | 0.002 |
| NMI | chr2:152145930-152148430 | NM_004688 | >0.05 | -0.01 | >0.05 | -0.081 |
| PCDH7 | chr4:30720036-30722536 | NM_032457 | <0.05 | -0.029 | >0.05 | 0.054 |

Abbreviations: LGG, lower-grade glioma; GBM, glioblastoma.


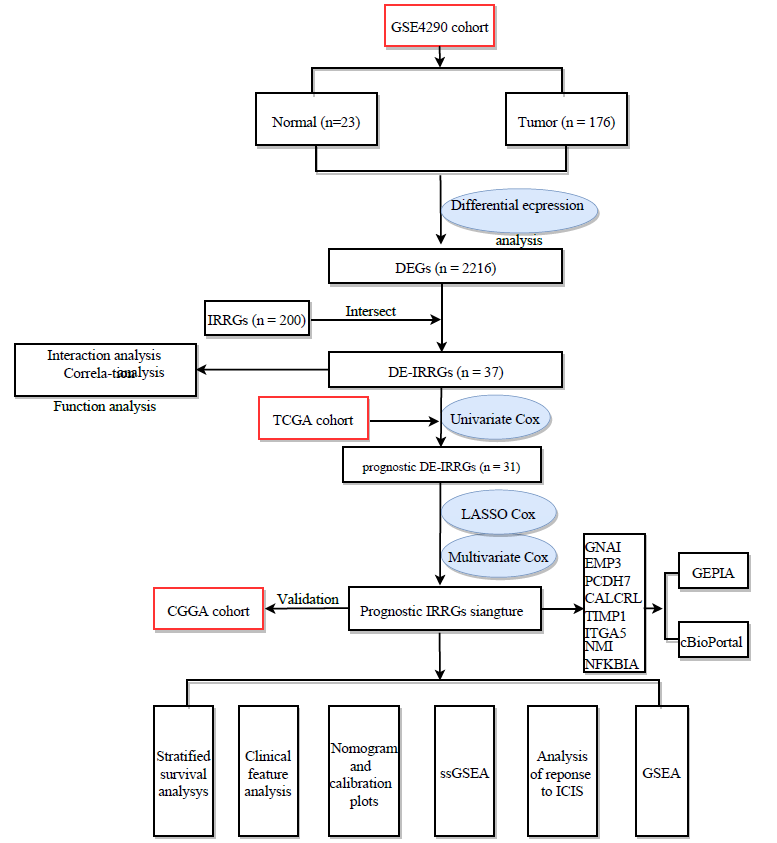


Supplemental Figure 1. Flow chart of the study design.


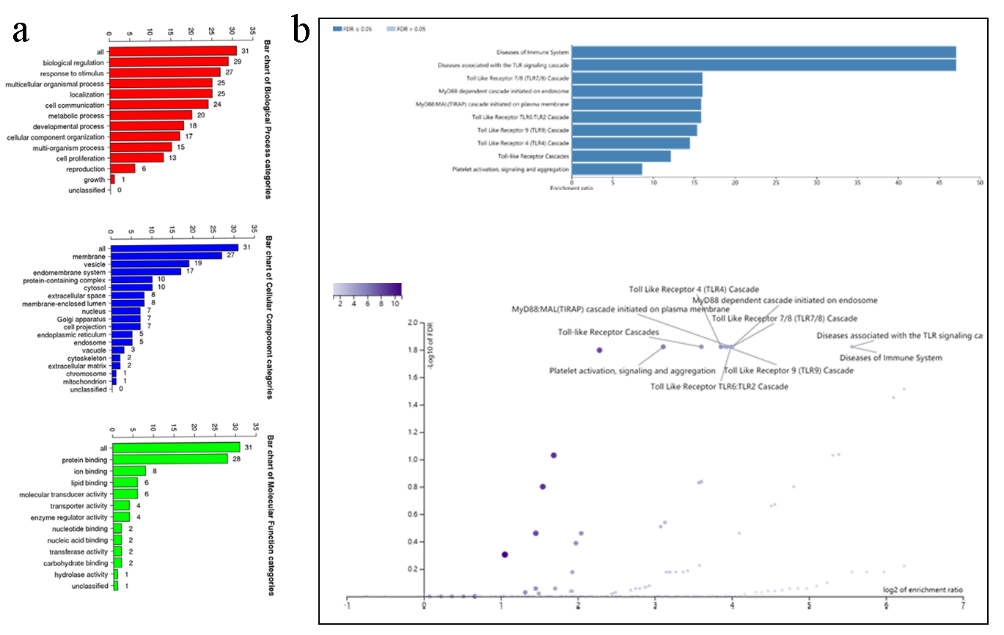


Supplemental Figure 2. Functional analysis for 31 differentially expressed inflammatory response-related genes using the WebGestalt database. (A) Gene Ontology (GO) enrichment analysis. GO terms consist of molecular functions (MF), biological processes (BP), and cellular components (CC). (B) Reactome pathway analysis.


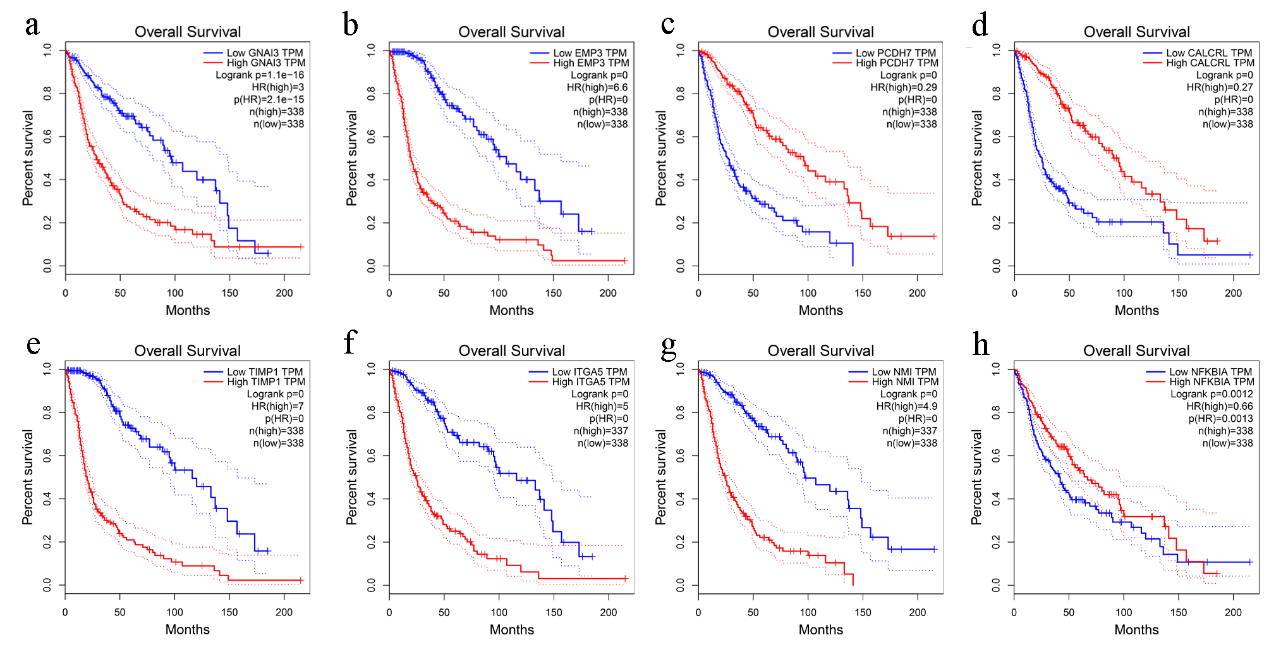


Supplemental Figure 3. The prognostic analysis of eight target IRRGs in the signature using the GEPIA database. (A) GNAI. (B)EMP3. (C) PCDH7. (D) CALCRL. (E) TIMP1. (F)ITGA5. (G)NMI. (H) NFKBIA.


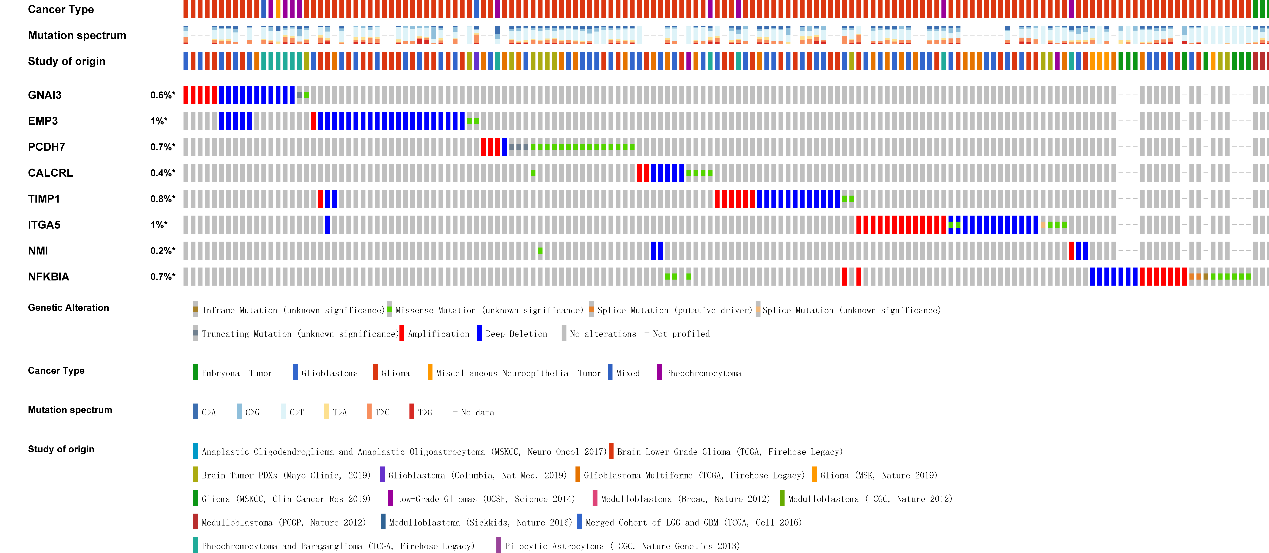


Supplemental Figure 4. The oncoprint plot describes the mutations, copy number variations, and structural variant landscape for the eight target IRRGs in glioma.


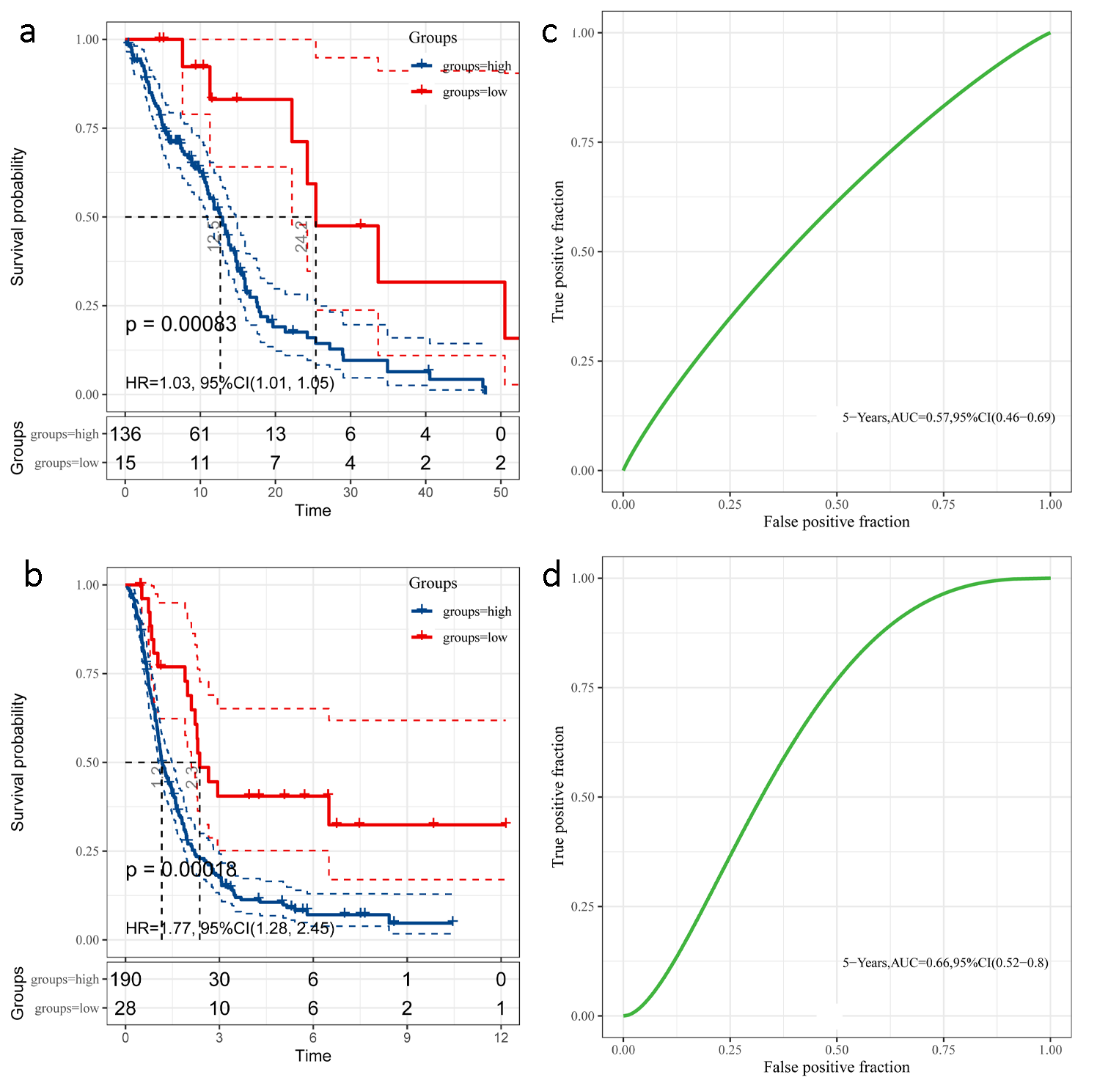


Supplemental Figure 5. Prognostic analysis for the IRRG signature in GBM patients in both TCGA and CGGA cohorts. (a) Kaplan-Meier curve for OS prediction in GBM patients in TCGA cohort. (b) The prognostic value of the IRRG signature at 5 years via ROC curve analysis in TCGA cohort. (c) Kaplan-Meier curve for OS prediction for GBM patients in the CGGA cohort. (d) The prognostic value for the IRRG signature at 5 years via ROC curve analysis in the CGGA cohort.


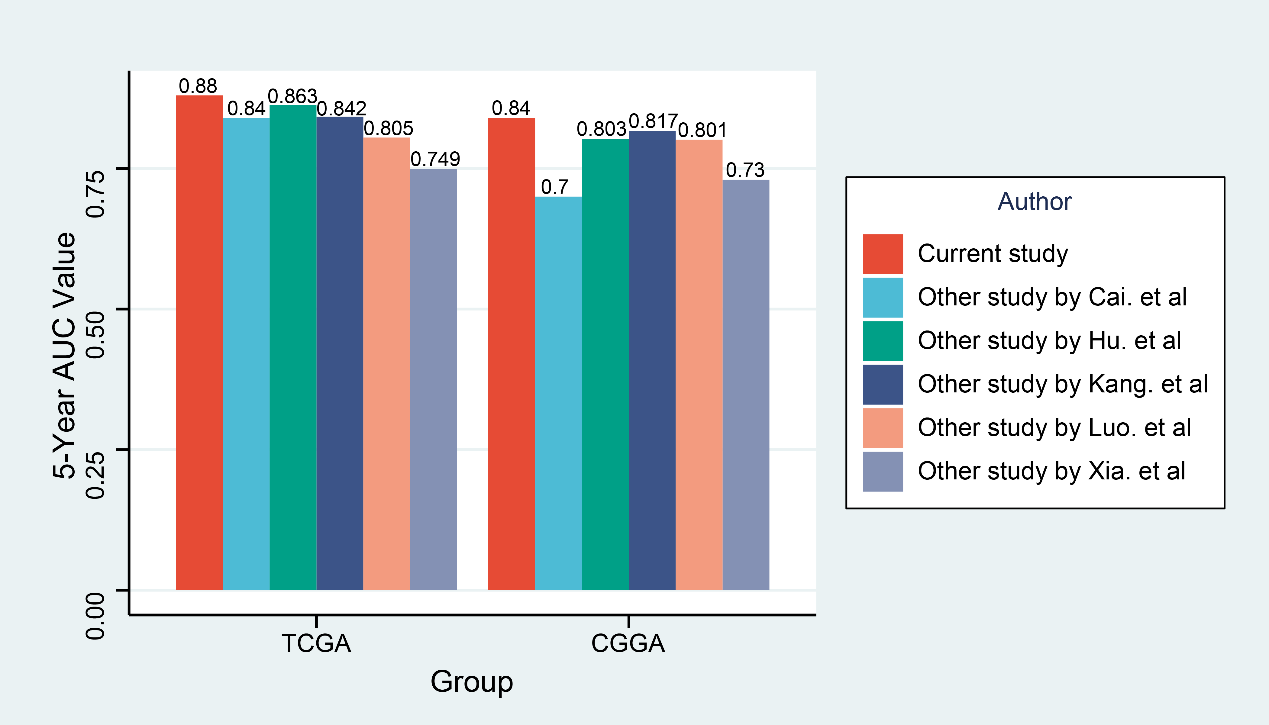


Supplemental Figure 6. Time-dependent ROC analysis for comparing the six signatures (including the IRRG signature of the present study) in predicting 5-year OS in both TCGA and CGGA databases.


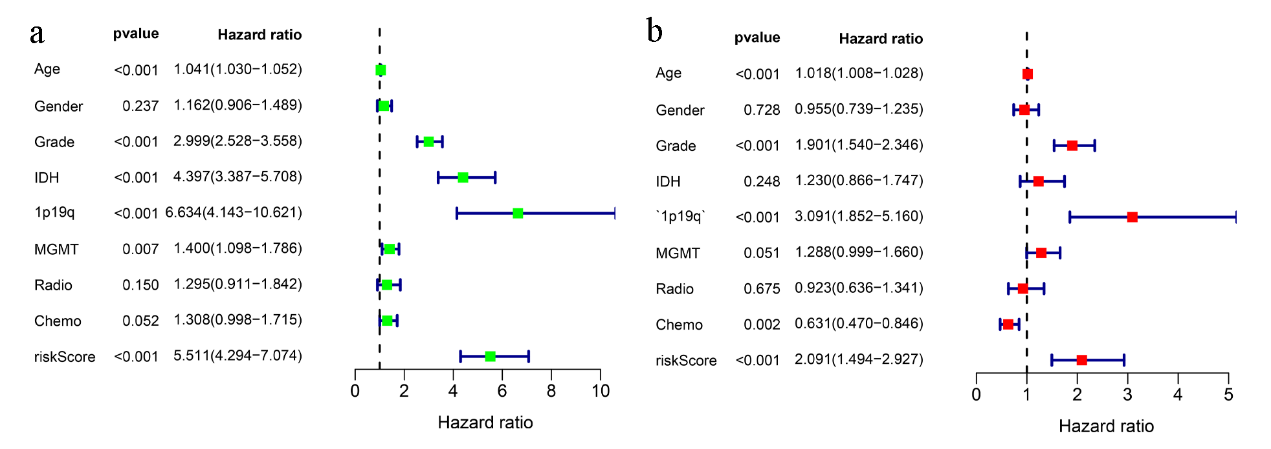


Supplemental Figure 7. Univariate (a) and multivariate (b) Cox regression analyses of the IRRG signature and clinical factors.
